# GABA_A_ receptor subunit gene polymorphisms predict symptom-based and developmental deficits in Chinese Han children and adolescents with autistic spectrum disorders

**DOI:** 10.1038/s41598-017-03666-0

**Published:** 2017-06-12

**Authors:** Shuhan Yang, Xuan Guo, Xiaopeng Dong, Yu Han, Lei Gao, Yuanyuan Su, Wei Dai, Xin Zhang

**Affiliations:** Department of Maternal, Child and Adolescent Health, School of Public Health, Tianjin Medical University, Tianjin, 300070 China

## Abstract

GABA_A_ receptor subunit genes *GABRB3, GABRA5*, and *GABRG3* located on chromosome 15q11-q13 have been implicated in the etiology of autistic spectrum disorders (ASD). This study intended to investigate the possible role of single-nucleotide polymorphisms (SNPs) present in *GABRB3* (rs2081648 and rs1426217), *GABRA5* (rs35586628), and *GABRG3* (rs208129) genes in ASD susceptibility and symptom-based and developmental phenotypes of ASD in Chinese Han children and adolescents. 99 ASD patients and 231 age- and gender- frequency-matched typical developing (TD) controls were tested by TaqMan® genotyping assay. Symptom-based phenotypes were evaluated by Childhood Autism Rating Scale (CARS) and Autism Behavior Checklist (ABC), and developmental phenotypes were assessed by Early Childhood Development Questionnaire (ECDQ) in ASD patients. Three haplotypes and global *χ* ^*2*^ test of all SNPs demonstrated significant associations between ASD and TD groups. Besides, *GABRB3* rs2081648, *GABRA5* rs35586628, and *GABRG3* rs208129 polymorphisms were associated with symptom-based deficits in social interaction, sensorimotor and somatosensory coordination, visual response, imitation, activity level, language expression and adaptability. Developmental abnormalities in late emergences of social interaction and fine motor were detected in *GABRB3* rs2081648 polymorphism. Overall results indicated that gene synergy may participate in ASD pathogenesis, and GABA_A_ receptor gene polymorphisms can predict symptom-based and developmental deficits in ASD individuals.

## Introduction

Autistic spectrum disorders (ASD) are a constellation of heterogeneous neurodevelopmental disorders characterized by early-onset deficits in social communication and interaction, and by restricted and repetitive behaviors, interests, or activities^1^. It is reported that the prevalence of ASD is on rise from 1/2000 in 1970s to 1/200 in 2000 s^2^ and a recent survey indicated that approximately 1 in 68 children has ASD, with a male: female ratio of 4.5:1^3^. ASD are typically considered as a group of life-long disorders, with heavy care and financial burdens on families and society. Nevertheless, there are still no doubtless pharmacological treatments to alleviate the core deficits in individuals with ASD. The obtainment of better targeted, ASD-specific therapies will only be possible according to a better understanding of ASD pathogenesis.

Genetics has a key role in the etiology of ASD, in conjunction with developmentally early environmental factors. However, these effects have not yet been confirmed. Emerging evidence suggests that increased excitatory and reduced inhibitory neurotransmission may form a final common pathway in ASD^4^. Brain hyperexcitability with abnormalities in GABA transmission^5, 6^ and lower brain gamma-aminobutyric acid (GABA) levels^7–9^, as well as reduced gamma-aminobutyric acid type A (GABA_A_) receptors in the superior frontal cortex of ASD patients^10^ have been assumed to underlie the symptoms of ASD.

GABA is the predominant inhibitory neurotransmitter in the adult brain, mainly acting via an intricate series of ionotropic GABA_A_ receptors (ligand-gated chloride channels) on the postsynaptic neuron. The GABA_A_ receptors are composed of 19 different subunits (α1–6, β1–3, γ1–3, δ, ε, θ, π, ρ1–3) arranged around a central pore and mediate the majority of fast synaptic inhibition in the brain. Activation of GABA_A_ receptors is associated with impaired long-term potentiation (LTP) and impaired learning *in vivo* ^11^. Although the GABA_A_ receptors contain the sites for several therapeutic drugs and agents, such as benzodiazepines, steroids, and anesthetics^12^, evidence supporting the benefit of GABAergic drugs in ASD is inconclusive and limited. GABA_A_ receptor subtypes may offer the promise of a new CNS pharmacology beyond classical benzodiazepines on the basis of the regulation of cognitive behavior by α5 GABA_A_ receptors^13^.

Converging genetic evidence specifically implicate the involvement of a cluster of GABA_A_ receptor subunit genes (*GABRB3, GABRA5 and GABRG3*) located on the 15q11-q13 (β3, α5 and γ3 subunits) region in the pathogenesis of ASD^14, 15^. These three GABA_A_ receptor subunit genes are physically positioned in the region on chromosome 15q which is most commonly reported loci of chromosomal abnormalities documented in patients with ASD^16, 17^, including deletions and duplications of chromosome 15q11-q13. For instance, maternal deletion of 15q11-q13 is responsible for Angelman syndrome which is characterized by impaired language and speech development, movement disorder, and mental retardation, while paternal deletion of 15q11.2-q12 is related to Prader-Willi syndrome which is characterized by hypotonia, short stature, and obesity. Both Angelman and Prader-Willi syndromes are liable to have ASD^18^.

Recently, both genomic linkage screens^19–23^ and linkage disequilibrium (LD)^24–29^ analyses have implicated *GABRB3* as an excellent candidate gene for ASD. Nevertheless, the association between *GABRB3* and ASD has not been universally identified^30–34^. Symptom-based phenotypes of ASD have offered some evidence for association with the *GABRB3* region. Affected individuals with ASD having high insistence-on-sameness scores exhibited a higher linkage signal to the *GABRB3* region among families^21^. *GABRB3* deficient mice showed occasional epilepsy, hyper-responsive to human contact, diminished nurturing behaviors, hyperactive run in tight circles, poor motor skills, electroencephalographic abnormalities, impaired social and exploratory behaviors, hypoplasia of cerebellar vermis and learning and memory deficits^35–40^. However, the association between the *GABRB3* region and savant skills is still contradictory with the positive^41^ and negative^42^ findings in ASD patients.

In addition to evidence implicating the *GABRB3* region, there exists relative fewer supports for association between ASD and the *GABRA5* region^27, 43^, as well as between ASD and the *GABRG3* region^44^. Most findings were negative in the *GABRA5* and *GABRG3* regions with ASD^15, 28, 32, 34, 45, 46^. However, Kim *et al*.^46^ found a nominally significant association between one single-nucleotide polymorphism (SNP) of *GABRA5* and a symptom-based phenotype defined as ‘relative failure to initiate or sustain conversational interchange’ in the form of inflexible language behavior^47^. Moreover, *GABRA5* deficient mice exhibited deficits in short-term memory when the task became increasingly more difficult. Reduced expression and function of *GABRA5* may cause neurodevelopmental changes that contribute to ASD-like behaviors^48^.

There exists most family-based association analysis studies of GABA_A_ receptor genes on chromosome 15q11-q13 for reports of the positive association between maternal interstitial duplication and ASD, as well as significant associations and linkage studies in chromosomally normal ASD families^41^, but case-control studies are rare and little is known about the degree to which genetic polymorphisms underlie symptom-based and developmental variability in ASD patients. To replenish ongoing efforts to describe allele associations at *GABRB3*, *GABRA5* and *GABRG3* in ASD family trios, we sought to utilize a case-control association study to observe relevant clues of ASD pathogenesis, as well as analyzing the associations between the *GABRB3* (rs2081648 and rs1426217), *GABRA5* (rs35586628), and *GABRG3* (rs208129) gene polymorphisms and symptom-based and developmental deficits of ASD patients in Chinese Han children and adolescents.

## Results

### Association analysis of *GABRB3*, *GABRA5*, and *GABRG3* SNPs

No significant deviations from the Hardy-Weinberg equilibrium were found in both ASD patients and typical developing (TD) controls for four SNPs, including two *GABRB3* SNPs (rs2081648 and rs1426217), one *GABRA5* SNP (rs35586628), as well as one *GABRG3* SNP (rs208129). The genotypic and allelic frequencies of the four SNPs between the ASD patients and TD controls demonstrated no statistical differences (*p* > 0.05) (Table 1).

**Table 1:** Genotypic and allelic frequencies of four SNPs in three GABA_A_ receptor genes and association analysis between the ASD and TD groups.

Gene	dbSNP ID	Chromsome Position	Group	Allele counts (frequency)		*p* Allele	Genotype counts (frequency)			*p* HWE-ASD	*p* HWE-TD	*p* Genotype
GABRB3	rs2081648	26553052		T	C	0.833	TT	TC	CC	0.815	0.214	0.529
			ASD	72 (0.36)	126 (0.64)		14 (0.14)	44 (0.45)	41 (0.41)			
			TD	172 (0.37)	290 (0.63)		27 (0.12)	118 (0.51)	86 (0.37)			
*GABRB3*	rs1426217	26575978		G	A	0.404	GG	GA	AA	0.123	0.724	0.091
			ASD	31 (0.16)	167 (0.84)		0 (0.00)	31 (0.31)	68 (0.69)			
			TD	61 (0.13)	401 (0.87)		5 (0.02)	51 (0.22)	175 (0.76)			
*GABRA5*	rs35586628	26886993		C	T	0.165	CC	CT	TT	0.341	0.719	0.310
			ASD	78 (0.39)	120 (0.61)		18 (0.18)	42 (0.42)	39 (0.40)			
			TD	209 (0.45)	253 (0.55)		49 (0.21)	111 (0.48)	71 (0.31)			
*GABRG3*	rs208129	27179760		T	A	0.314	TT	TA	AA	0.252	0.084	0.634
			ASD	95 (0.48)	103 (0.52)		26 (0.26)	43 (0.44)	30 (0.30)			
			TD	202 (0.44)	260 (0.56)		51 (0.22)	100 (0.43)	80 (0.35)			

All four SNPs locate in intron regions. ASD, autistic spectrum disorders; HWE, Hardy-Weinberg equilibrium; SNP, single-nucleotide polymorphism; TD, typical developing.

Linkage disequilibrium (LD) analysis showed two *GABRB3* SNPs (rs2081648 and rs1426217) were in strong disequilibrium in ASD patients (*D*′ = 0.795) and in positive disequilibrium in TD controls (*D′* = 0.532). Four SNPs of *GABRB3* (rs2081648 and rs1426217), *GABRA5* (rs35586628), and *GABRG3* (rs208129) formed eleven effective haplotypes in both ASD and TD groups, and three haplotypes were associated with ASD (Table 2). The global *p* was 0.007 (*χ* ^*2*^ = 24.33, *df* = 10) among four SNPs between ASD and TD groups.

**Table 2:** Haplotype analysis for the genetic association of *GABRB3*, *GABRA5*, and *GABRG3* between ASD patients and TD controls.

Haplotypes	ASD patients counts (frequency)	TD controls counts (frequency)	*χ* ^2^	*p*	ORs (95% CI)
CACA	28.99 (0.15)	61.49 (0.13)	0.08	0.772	1.07 (0.66–1.73)
CACT	18.41 (0.09)	55.84 (0.12)	1.40	0.237	0.72 (0.41–1.25)
CATA	28.05 (0.14)	88.15 (0.19)	2.91	0.088	0.67 (0.42–1.06)
CATT	46.70 (0.24)	65.47 (0.14)	7.61	0.006**	1.80 (1.18–2.74)
TACA	7.92 (0.04)	43.66 (0.09)	6.24	0.013*	0.38 (0.18–0.83)
TACT	9.78 (0.05)	23.34 (0.05)	0.02	0.876	0.94 (0.44–2.03)
TATA	16.36 (0.08)	27.26 (0.06)	1.01	0.315	1.39 (0.73–2.62)
TATT	10.79 (0.05)	35.79 (0.08)	1.36	0.244	0.66 (0.33–1.33)
TGCA	9.06 (0.05)	6.74 (0.02)	5.38	0.020*	3.13 (1.14–8.60)
TGTA	10.61(0.05)	18.56 (0.04)	0.46	0.500	1.30 (0.60–2.83)
TGTT	7.48 (0.04)	11.16 (0.02)	0.80	0.372	1.53 (0.60–3.93)

Haplotypes with a frequency of < 0.03 in both groups have been dropped. Haplotypes were constructed with rs2081648-rs1426217-rs35586628-rs208129 in proper order. *p < 0.05; **p < 0.01. ASD, autistic spectrum disorders; CI, confidence interval; ORs, odds ratios; TD, typical developing.

### *GABRB3*, *GABRA5*, and *GABRG3* SNPs and ASD symptom-based phenotypes

For the CARS total scores deviated from a normal distribution (*p* < 0.05), we conducted a Kruskal-Wallis H test to analyze the relationships between CARS total scores and the genotypes of four SNPs, and found no evidence for any associations. Nevertheless, an ordinal polytomous logistic regression analysis showed some significant associations between ratings in items of CARS and genotypes of *GABRB3* SNP rs2081648, *GABRA5* SNP rs35586628, and *GABRG3* SNP rs208129 in ASD patients (while adjusting for sex, age, and IQ). ORs (95% CI) for the ‘4 = severely abnormal’ among different genotypes were shown in Table 3. ASD patients with a TT genotype of *GABRB3* SNP rs2081648 trended to suffer from severe abnormality of ‘visual response’ compared to those with a TC genotype. Moreover, ASD participants with CC and TT genotypes of *GABRA5* SNP rs35586628 were likely to perform worse in ‘verbal communication’ than those with a CT genotype, and ASD individuals with TA and TT genotypes of *GABRG3* SNP rs208129 showed worse performance in ‘imitative behavior’ and ‘activity level’ than those with an AA genotype.

**Table 3:** Odds ratios (ORs) with 95% confidence intervals (CI) of having severe abnormality in items of CARS among genotypes of *GABRB3* SNP rs2081648, *GABRA5* SNP rs35586628, and *GABRG3* SNP rs208129 in ASD patients.

Gene	dbSNP ID	Items of CARS	Ref	Comparisons between genotypes		
*GABRB3*	rs2081648			TT vs. TC	TT vs. CC	TC vs. CC
		Visual response	4	3.54 (1.03–12.11)*	2.07 (0.97–4.41)	0.58 (0.32–1.06)
*GABRA5*	rs35586628			CC vs. CT	CC vs. TT	CT vs. TT
		Verbal communication	4	3.80 (1.09–13.29)*	1.98 (0.92–4.24)	0.52 (0.28–0.96)*
*GABRG3*	rs208129			AA vs. TA	AA vs. TT	TA vs. TT
		Imitative behavior	4	0.35 (0.12–0.99)*	0.49 (0.25–0.95)*	1.40 (0.79–2.48)
		Activity level	4	0.14 (0.04–0.59)**	0.36 (0.14–0.90)*	2.49 (1.24–4.99)*

Ratings in items of CARS with a four-point scale (1 = appropriate for age; 2 = mildly abnormal; 3 = moderately abnormal; 4 = severely abnormal). The reference (Ref) rating is 4 in an ordinal polytomous logistic regression analysis (while adjusting for sex, age, and IQ). ASD patients for *GABRB3* rs2081648: *n* = 14 TT, *n* = 44 TC, and *n* = 41 CC. ASD patients for *GABRA5* rs35586628: *n* = 18 CC, *n* = 42 CT, and *n* = 39 TT. ASD patients for *GABRG3* rs208129: *n* = 26 TT, *n* = 43 TA, and *n* = 30 AA. *p < 0.05; **p < 0.01. ASD, autistic spectrum disorders; CARS, Childhood Autism Rating Scale; Ref, reference; vs., versus.

Scores of each ABC category and ABC total scores conformed to a normal distribution and homogeneity of variance (*p* > 0.05), but no significant associations between genotypes of four SNPs and all ABC scores were observed after the one-way ANCOVA with sex, age, and IQ as covariates (*p* > 0.05). However, a binary logistic regression analysis found some significant associations between ratings in items of ABC and genotypes of *GABRB3* SNP rs2081648, *GABRA5* SNP rs35586628, and *GABRG3* SNP rs208129 in ASD patients (while adjusting for sex, age, and IQ). ORs (95% CI) for ‘1 = yes’ of existing relevant symptoms among different genotypes were shown in Table 4.

**Table 4:** Odds ratios (ORs) with 95% confidence intervals (CI) of existing relevant impairments in items of ABC among genotypes of *GABRB3* SNP rs2081648, *GABRA5* SNP rs35586628, and *GABRG3* SNP rs208129 in ASD patients.

Gene	dbSNP ID	Items of ABC	Ref	Comparisons between genotypes		
*GABRB3*	rs2081648			TT vs. CC	TC vs. CC	TC vs. TT
		(R) Has not developed any friendships	1	0.70 (0.16–3.12)	0.33 (0.12–0.93)*	0.48 (0.12–1.93)
*GABRA5*	rs35586628			CC vs. TT	CT vs. TT	CT vs. CC
		(R) Does not reach out when reached for	1	0.48 (0.09–2.61)	3.29 (1.14–9.48)*	6.86 (1.33–35.40)*
		(S) Sometimes shows no ‘startle response’ to loud noise	1	3.02 (0.86–10.63)	4.18 (1.53–11.41)**	1.39 (0.43–4.51)
		(L) Cannot point to more than five named objects	1	2.64 (0.80–8.78)	0.61 (0.23–1.58)	0.23 (0.07–0.78)*
		(L) Repeats sounds or words over and over	1	1.59 (0.48–5.29)	3.54 (1.38–9.04)**	2.23 (0.70–7.17)
*GABRG3*	rs208129			AA vs. TT	TA vs. TT	TA vs. AA
		(s′) Strong reactions to changes in routine/environment	1	3.01 (0.79–11.54)	5.09 (1.46–17.77)*	1.69 (0.60–4.78)
		(S) Sometimes painful stimuli such as bruises, cuts, and injections evoke no reaction	1	10.61 (1.21–92.92)*	14.42 (1.73–120.46)*	1.36 (0.49–3.76)
		(L) Uses at least 15 but less than 30 spontaneous phrases daily to communicate	1	0.29 (0.09–0.94)*	0.98 (0.32–2.97)	3.34 (1.21–9.22)*

Ratings in items of ABC with a two-point scale (0 = no; 1 = yes). The reference (Ref) rating is 1 in a binary logistic regression analysis (while adjusting for sex, age, and IQ). ASD patients for *GABRB3* rs2081648: *n* = 14 TT, *n* = 44 TC, and *n* = 41 CC. ASD patients for *GABRA5* rs35586628: *n* = 18 CC, *n* = 42 CT, and *n* = 39 TT. ASD patients for *GABRG3* rs208129: *n* = 26 TT, *n* = 43 TA, and *n* = 30 AA. *p < 0.05; **p < 0.01. ABC, Autism Behavior Checklist; ASD, autistic spectrum disorders;  L, language; R, relating; S, sensory; s′, social and self-help; SNP, single-nucleotide polymorphism; Ref, reference; vs., versus.

### *GABRB3*, *GABRA5*, and *GABRG3* SNPs and developmental phenotypes of ASD

An ordinal polytomous logistic regression analysis detected significant associations between ratings in two items of ECDQ and genotypes of *GABRB3* rs2081648 in ASD patients (while adjusting for sex, age, and IQ) (*n* = 9 TT, *n* = 31 TC, and *n* = 27 CC). The OR for ‘3 = late’ of ‘smiling to his/her mother’ in those with a TT genotype compared to those with a CC genotype was 4.39 (95% CI = 1.43–13.52, *p* = 0.010), those with a TC genotype compared to those with a CC genotype was 0.21 (95% CI = 0.09–0.48, *p* = 0.000), and those with a TT genotype compared to those with a TC genotype was 20.86 (95% CI = 3.39–128.50, *p* = 0.001). We found that ASD patients with a TT genotype trended to show social interaction in older ages. In addition, the OR for ‘3 = late’ of ‘grasping things by himself/herself’ in those carrying a TT genotype compared with those carrying a CC genotype was 2.26 (95% CI = 0.86–5.91, *p* = 0.098), those carrying a TC genotype compared with those carrying a CC genotype was 0.28 (95% CI = 0.13–0.63, *p* = 0.002), and those carrying a TT genotype compared with those carrying a TC genotype was 8.02 (95% CI = 1.59–40.56, *p* = 0.012). As a result, ASD participants with a TC genotype developed earlier in fine motor.

## Discussion

To our knowledge, this is the first age- and gender- frequency-matched case-control study designed to investigate the association between the three GABA_A_ receptor genes, *GABRB3*, *GABRA5*, and *GABRG3*, and ASD in Chinese Han children and adolescents, as well as to detect relevant symptom-based and developmental deficits associated with the three GABA_A_ receptor gene polymorphisms in Chinese Han children and adolescents with **A**SD.

Previous studies have investigated the association of the three GABA_A_ receptor genes with ASD, but the contradictory findings demand subsequent replications in different populations. In this study, we found no positive evidence of the associations between any single SNP of the three GABA_A_ receptor genes and ASD. Similar to our result, McCauley *et al*.^27^ demonstrated the negative evidence for the associations between three SNPs (rs2081648, rs1426217, and rs208129) and ASD, as well as Kim *et al*.^46^ reported no significant supports for four SNPs (rs2081648, rs1426217, rs35586628, and rs208129) of ASD mainly in Caucasian population with family trios. However, Kim *et al*.^29^ found an allele at SNP rs2081648 exhibited prior transmission in Korean trios. These differences may originate from the multiple demographic characteristics of the ASD populations (age, gender, ethnicity, sample size, diagnostic criteria, etc.) and different investigative methods (family-based association, case-control association, etc). The statistical power of our case-control association study is approximately 0.65 (*α* = 0.05) under the assumptions of 0.28% prevalence of ASD in Tianjin of China^49^ and genotypic relative risk of 1.8^34^. Thus small effect might not be detected because of the sample size.

Moreover, we detected the TD controls had lower *D′* than ASD patients, which predicted that TD individuals might have more chance to restructure randomly in accordance with genetic equilibrium than ASD individuals. Further studies can apply family-based association test (FBAT) in Chinese Han family trios of ASD and TD children and adolescents with the three GABA_A_ receptor genes to discover more significant evidence on this difference. In addition, our study revealed that individuals with a CATT haplotype and a TGCA haplotype were more likely to have ASD, but individuals with a TACA haplotype had more potential to be TD controls. Our findings supported that the etiology of ASD may include gene-gene interactions rather than the effect of one single gene^50^.

For the limited evidence on the association between GABA_A_ receptor genes and characteristic phenotypes of ASD, we further observed some significant associations between three SNPs of the three GABA_A_ receptor genes and phenotypic features of ASD without *GABRB3* SNP rs1426217. In the symptom-based phenotypes, as a supplement for the detections of associations between *GABRB3* region and high degree of insistence-on-sameness^21^, as well as savant skills^41^ in previous studies, the ASD patients in our study with a TT genotype of *GABRB3* SNP rs2081648 trended to perform worse in ‘visual response’ of CARS, and those with a CC genotype of *GABRB3* SNP rs2081648 were more likely to show a deficit in social interaction according to the ‘relating’ part of ABC (38 = (R) Has not developed any friendships). Moreover, ASD patients with a CC genotype of *GABRA5* SNP rs35586628 exhibited a deficit in language expression or cognitive functioning basing on the ‘verbal communication’ of CARS and the ‘language’ part of ABC (37 = (L) Cannot point to more than five named objects), and the others with a CT genotype showed sensorimotor and somatosensory abnormalities (13 = (R) Does not reach out when reached for; 29 = (S) Sometimes shows no ‘startle response’ to loud noise), and rigid language patterns (48 = (L) Repeats sounds or words over and over) according to the ‘relating’, ‘sensory’ and ‘language’ parts of ABC. Our finding provided more supports for the relationships between genotypes of *GABRA5* and core features of ASD than Kim *et al*.^46^, who only reported a significant association between one *GABRA5* SNP rs2075716 and ‘relative failure to initiate or sustain conversational interchange’. Furthermore, the ASD patients with a TA genotype of *GABRG3* SNP rs208129 showed the highest potential to bear the most severe deficits in ‘imitative behavior’ and ‘activity level’ of CARS, as well as to display insistence-on-sameness or worse adaptability (14 = (s′) Strong reactions to changes in routine/environment), somatosensory disability (26 = (S) Sometimes painful stimuli such as bruises, cuts, and injections evoke no reaction) and difficulties in social communication (56 = (L) Uses at least 15 but less than 30 spontaneous phrases daily to communicate) in the ‘social and self-help’, ‘sensory’ and ‘language’ parts of ABC. Above all, the heterozygotes of *GABRA5* SNP rs35586628 and *GABRG3* SNP rs208129 showed the primary potential to increase the probability for ASD patients to undertake relevant core deficits, learning disabilities, and especially the decreased sensory sensitivity in voice and pain conformed abnormal sensory processing as one of the core features of individuals with ASD^1^. In the developmental phenotypes, ASD patients with a TT genotype of *GABRB3* SNP rs2081648 were likely to show negative effects on initial social interaction and fine motor with the late emergences of ‘smiling to his/her mother’ and ‘grasping things by himself/herself’. As far as we know, there has been no previous studies intending to distinguish different phenotypes of early childhood development based on SNPs in children and adolescents with ASD. However, during the fetus period, the GABA_A_ receptor subunit cluster (containing *GABRB3*, *GABRA5*, and *GABRG3*) on chromosome 15q11-q13 region acts as a developmental role in GABAergic signaling for building neuronal connectivity, and plays a pivotal role in the maintenance of inhibitory tone in the adult brain^41^. Therefore, any abnormalities in the three genes are possible to cause developmental deficits in the whole life. Subsequent researches should pay more attention to the associations between polymorphisms of GABA_A_ receptor genes and early childhood development in both ASD patients and TD controls to obtain better and precise clues of early diagnosis for ASD patients, so as to intervene at an earlier time.

In conclusion, our findings are supportive of the fact that different subunits of GABA_A_ receptor subtypes may work together in the pathogenesis of ASD, as well as play differential roles in the symptom-based and developmental deficits in Chinese Han children and adolescents with ASD. To date, very few studies have explored the role of these genetic variants of *GABRB3*, *GABRA5*, and *GABRG3* genes in both ASD susceptibility and phenotypes, replication studies with a larger sample size are required to confirm our findings in different races.

## Materials and Methods

### Participants

The study protocol was approved by the Tianjin Medical University Institutional Review Board (Ethics Committee of Tianjin Medical University) and written informed consent was obtained from school principals, parents and/or caregivers to allow the collection and genetic analysis of DNA samples after a complete and extensive description of the study, in accordance with the Declaration of Helsinki. All participants enrolled into this study were Chinese Han children and adolescents from Tianjin, China. We recruited 99 ASD patients (79 males and 20 females) from five local special education schools and 231 age- and gender- frequency-matched TD controls (185 males and 46 females) from four local mainstream schools with a male to female ratio of 4:1 between 2 and 18 years of age. The diagnosis of ASD was made by qualified and experienced psychiatrists basing on the criteria of Diagnostic and Statistical Manual of Mental Disorders, Fourth Edition (DSM-IV)^51^ and was further identified using the Childhood Autism Rating Scales (CARS)^52^. A CARS total score of ≥ 30 is the cutoff for distinguishing children ‘at risk’ for autism from pervasive developmental disorder-not otherwise specified (PDD-NOS)^53, 54^, and a CARS total score of ≥ 25.5 is indicative of ASD^54^. Meanwhile, the Autism Behavior Checklist (ABC)^55^ was also administered for the assessment of their symptom-based phenotypes and Early Childhood Development Questionnaire (ECDQ) was used to evaluate their developmental phenotypes. In addition, intelligent quotient (IQ) was determined using the Chinese Binet Scale (Binet)^56^ by clinical pediatric psychologists. ASD subjects were included with a full-scale IQ of ≥ 30 and TD controls were included with an IQ in the average range. Participant characteristics are presented in Table 5. In the present study, none of the participants in any group were taking drugs. Exclusion criteria for both groups included medical history of epileptic seizures, head trauma, and known psychiatric, neurological or genetic disorders, and TD controls were with a score of < 7 on the Clancy Autism Behavior Scale (CABS)^57^.

**Table 5:** Participant characteristics.

Participants	Total (*n*)	Sex (*n* (frequency))		Age (year)	CARS total score	Full-scale IQ
		Male	Female			
ASD group	99	79 (0.80)	20 (0.20)	8.53 ± 0.42	33.30 ± 0.45	56.21 ± 1.50
﻿﻿﻿﻿﻿ Autism	82	64 (0.78)	18 (0.22)	8.95 ± 0.47	34.43 ± 0.45	52.16 ± 1.26
﻿﻿ Severe autism	25	20 (0.80)	5 (0.20)	10.28 ± 0.82	39.88 ± 0.43	43.08 ± 1.83
Mild/moderate autism	57	44 (0.77)	13 (0.23)	8.37 ± 0.56	32.04 ± 0.22	56.14 ± 1.32
PDD-NOS	17	15 (0.88)	2 (0.12)	6.47 ± 0.76	27.88 ± 0.27	75.76 ± 3.61
TD group	231	185 (0.80)	46 (0.20)	8.44 ± 0.25		102.07 ± 0.51

The values are expressed in mean ± SEM. ASD, autistic spectrum disorders; CARS, Childhood Autism Rating Scale; IQ, intelligent quotient; n, number; PDD-NOS, pervasive developmental disorder-not otherwise specified, SEM, standard error of the mean; TD, typically developing.

### Phenotypic Assessment

#### Childhood Autism Rating Scale (CARS)

The Childhood Autism Rating Scale (CARS) is a 15-item behavior-based clinical evaluation that includes 14 domains plus one category of general impressions rated on a four-point scale (1 = appropriate for age; 2 = mildly abnormal; 3 = moderately abnormal; 4 = severely abnormal) according to interaction and observation. The 15 items are as follows: ‘relating to people’, ‘imitative behavior’, ‘emotional response’, ‘body use’, ‘object use’, ‘adaptation to change’, ‘visual response’, ‘listening response’, ‘perceptive response’, ‘fear or anxiety’, ‘verbal communication’, ‘non-verbal communication’, ‘activity level’, ‘level and consistency of intellective relations’, and ‘general impressions’^55^. The CARS has been proved to have a high degree of internal consistency, inter-rater and test-retest reliability, good discriminant validity, and high criterion-related validity^58^. It is widely used by psychiatrists to identify children with autism and as a further measurement of the severity of this disease. Total scores can range from a low of 15 to a high of 60; scores of less than 30 indicate that the individual is in the non-autistic range, scores between 30 and 36.5 indicate mild/moderate autism, and scores from 37 to 60 indicate severe autism^53^. In this study, all ASD patients got this assessment.

#### Autism Behavior Checklist (ABC)

The Autism Behavior Checklist (ABC) is a behavior checklist that consists of 57 items in 5 categories: ‘sensory (S) (9 items)’, ‘relating (R) (12 items)’, ‘body and object use (B) (12 items)’, ‘language (L) (13 items)’, and ‘social and self-help (s′) (11 items)’. Each item corresponds to a single score referring to a single symptomatological area^55^. For each item, we set the ratings as a two-point scale (0 = no; 1 = yes). The scale utilizes an observer’s rating of a series of typical autistic behaviors in a certain subject and provides advice for educational intervention. In this study, all ASD patients got this assessment.

#### Early Childhood Development Questionnaire (ECDQ)

The initial ages of early childhood development were assessed by parents or caregivers according to the normal range of age in different developmental parts of the ECDQ. It contains 9 items, including ‘smiling to his/her mother’, ‘grasping things by himself/herself’, ‘sitting by himself/herself’, ‘walking by himself/herself’, ‘calling daddy or mummy’, ‘speaking phrases’, ‘controlling defecate and urinate’, ‘stopping wetting the bed’, ‘wearing by himself/herself’. The developmental stage was evaluated on the ratings of a three-point scale (1: early, 2: normal, 3: late) for each participant. 67 parents or caregivers of ASD patients completed this assessment.

### Sample Collection

Participants were previously informed to fast in the morning before sampling to avoid the influence of food and/or drink. DNA samples were obtained by an experienced technician by taking a sterile swab and rubbing it against the inside of participants’ cheeks for 1 min and placing it into a labeled sterile 2-ml Falcon tube. This was done for both cheeks.

### DNA Extraction

DNA was extracted from the swabs using the Swab Gen DNA Kit (CW0530, CWBIO, Beijing, China) according to the manufacturer’s suggestion. The DNA solution for the current experiment was then stored at 4 °C, and the redundant stock was stored at −20 °C. The concentration of each DNA sample was determined using a NanoDrop^®^ ND-2000 spectrophotometer (Thermo Scientific).

### SNP Selection and Genotyping

Through the SNP database (http://www.ncbi.nlm.nih.gov/snp/), we selected four SNPs of the GABA_A_ receptor genes with relatively high allele frequency and heterozygosity in Asian populations, including two *GABRB3* SNPs (rs2081648, Assay ID: C___2911917_10; rs1426217, Assay ID: C___2901088_10), one *GABRA5* SNP (rs35586628, Assay ID: C____252720_10), as well as one *GABRG3* SNP (rs208129, Assay ID: C___2665692_10).

All SNPs were genotyped by TaqMan® genotyping assay. The TaqMan probes were ordered from the Assays on Demand system of the Applied Biosystems (Applied Biosystems, Foster City, CA, USA). Genotyping was performed in 96-well plates in 5-μl system containing 2.5 μl of TaqMan® Genotyping Master Mix (Applied Biosystems, Foster City, CA, USA), 0.125 μl of 40 × TaqMan probe (Applied Biosystems, Foster City, CA, USA), 1.375 μl ddH_2_O and 1 μl (5–20ng) of genomic DNA using Roche 480 PCR System (Roche Applied Science, Penzberg, Germany) in accordance with the manufacturer’s instructions. The first row of each 96-deep-well plate contained the negative controls, which consisted of 2.5 μl of TaqMan® Genotyping Master Mix (Applied Biosystems, Foster City, CA, USA), 0.125 μl of 40 × TaqMan probe (Applied Biosystems, Foster City, CA, USA) and 2.375 μl ddH_2_O. PCR parameters were as follows: enzyme activation: 95 °C for 10 min, 45 cycles of amplification: 95 °C for 15 s and 60 °C for 1 min and cooling at 40 °C for 10 s. Results from the amplified PCR products were viewed using the Roche LightCycler^®^480 II Real-Time PCR System (Roche Applied Science, Penzberg, Germany). Analysis of the SNP genotypes was performed using a Roche LightCycler^®^480 Sequence Detection System (Roche Diagnostics GmbH, Penzberg, Germany).

### Statistical Analysis

In both ASD patients and TD controls, Haploview version 4.2^59, 60^ was conducted to assess the Hardy-Weinberg equilibrium for genotypic frequency distributions by *χ* ^*2*^ test and the linkage disequilibrium (LD) analysis among SNPs by *D′*. A value of *D′* > 0.5 was defined as a positive linkage and *D′* > 0.75 were considered as a strong linkage. Power and Sample Size Calculation (http://biostat.mc.vanderbilt.edu/wiki/Main/PowerSampleSize) was employed to perform a power analysis. Haplotype counts and frequencies in both ASD and TD groups were evaluated using SHEsis (http://analysis.bio-x.cn/SHEsisMain.htm)^61^, a frequency of < 0.03 in both groups was ignored in the association analysis. The other analyses were conducted by SPSS version 17.0 (IBM Corporation New York, USA) and SAS version 9.3.2 (SAS Institute Inc, Cary, NC). Comparisons of genotypic and allelic frequencies between ASD patients and TD controls were performed with the *χ* ^*2*^ test. The Kolmogorov-Smirnov test was performed to check the distribution of our data. The Levene’s test was utilized to determine the homogeneity of variance. Kruskal-Wallis H tests were utilized for comparisons of CARS total scores among genotypes of four SNPs. A one-way analysis of covariance (ANCOVA) with sex, age, and IQ as covariates was applied for comparisons of ABC scores among genotypes of four SNPs. While adjusting for sex, age, and IQ, an ordinal polytomous logistic regression analysis was performed to discover the associations between ratings in items of CARS as well as ECDQ and genotypes of four SNPs, and a binary logistic regression analysis was utilized to find the associations between ratings in items of ABC and genotypes of four SNPs in ASD patients. Odds ratios (ORs) were expressed with 95% confidence intervals (95% CI). *Post-hoc* Bonferroni correction for multiple comparisons was performed. A *p* value of < 0.05 was described as significant.

### Data Availability

The datasets generated and analyzed during the current study are not publicly available due to the public availability would violate the privacy of participants, especially for the special individuals with ASD. Data are from the Department of Maternal, Child and Adolescent Health, School of Public Health, Tianjin Medical University and are available from the corresponding author (email: zhangxin@tmu.edu.cn) on a reasonable request.
